# Schwannoma at an Unusual Site: Case Report and Surgical Technique Discussion for Nasal Dorsum

**DOI:** 10.1155/2024/9422104

**Published:** 2024-09-16

**Authors:** Sultan K. Kadasah, Abdulrazaq M. Alshammari, Nader S. Alharbi, Ibtihal S. Alshehri, Raghad Y. Alasiri, Saud A. Aldhabaan, Ghalib X. Alsayed, Adnan Q. Almalki

**Affiliations:** ^1^ Department of Surgery College of Medicine University of Bisha, Bisha, Aseer, Saudi Arabia; ^2^ Department of Otorhinolayrngology Head and Neck Surgery Almaarefa University, Riyadh, Saudi Arabia; ^3^ Department of Otorhinolaryngology Armed Forces Hospital, Khamis Mushait, Aseer, Saudi Arabia

## Abstract

Peripheral nerve sheath tumors (PNSTs), while uncommon, can have a significant impact on appearance and quality of life, especially when they form in prominent areas such as the nose dorsum. We discuss a case of a 29-year-old woman who developed a benign PNST on the right side of her nasal ala. This tumor gradually grew, impairing her face appearance. Diagnostic tests, such as computed tomography (CT) and magnetic resonance imaging (MRI), revealed the tumor as a slowly growing, well-defined mass. The tumor was removed via open rhinoplasty under general anesthesia, and pathological investigation verified its benign nature. After surgery, the patient's quality of life improved significantly, and there were no evidence of tumor recurrence after eight months. This case emphasizes the need of including PNST in the differential diagnosis of nasal tumors.

## 1. Introduction

Peripheral nerve sheath tumors (PNSTs) are neoplasms that originate from Schwann cells, the primary glia of the peripheral nervous system. These tumors are classified as benign, malignant, or hybrid, with the latter having mixed histological features within the same mass [1–5]. Benign PNSTs are largely schwannomas and neurofibromas [2–4, 6–8], with malignant variations including neurofibrosarcoma, malignant schwannoma, and neurosarcoma [9–12]. PNSTs can occur spontaneously or as part of a hereditary condition such as schwannomatosis or neurofibromatosis types 1 and 2 [1–4].

In the head and neck, benign PNSTs are more common than malignant types, which are uncommon [6, 13]. Benign tumors, in particular, rarely impact the nasal dorsum and tip, which are considered uncommon for these neoplasms [14]. Although nasal PNSTs rarely cause symptoms, when they do, they are mainly caused by the tumor bulk [8]. In the situation addressed in our study, the patient's major concern was cosmetic, which led to the decision to do surgical excision along with open rhinoplasty. This method not only enhanced aesthetics but also improved functional outcomes and increased patient satisfaction after surgery [10].

Despite extensive study on PNSTs, significant gaps still exist, particularly in understanding the pathophysiological pathways that separate benign from malignant changes. Furthermore, the rarity of nasal placement in PNSTs complicates diagnosis and management, emphasizing the necessity for additional research into these unusual presentations. There is also a lack of awareness of the genetic and environmental factors that influence the development of hybrid PNSTs, as well as the clinical ramifications.

Our findings add to the limited literature on PNSTs in the nasal area, where these tumors are infrequently recorded. This study sheds light on the treatment and outcomes of a benign nasal PNST treated with surgical excision and rhinoplasty, providing insights into appropriate management options for such uncommon cases. The findings highlight the relevance of aesthetic considerations in surgical procedures for face malignancies, which can have a major impact on the patient quality of life. Furthermore, this study emphasizes the possibility of functional recovery and happiness, confirming the need for individualized surgical methods in similar situations. As a result, our study not only fills a knowledge gap but also lays the groundwork for future studies aimed at enhancing diagnosis accuracy and management success for PNSTs in locations that are unusual.

## 2. Case Presentation

We present the case of a 29-year-old female patient with an established diagnosis of hypothyroidism who has been treated with levothyroxine since the diagnosis. She came to the otolaryngology clinic with a five-year history of a right nasal mass. The mass has been gradually growing, remains painless, and nonpulsatile. The patient's primary concern was the disfiguring appearance of the mass, which had a negative impact on her quality of life. She reported no other symptoms, such as changes in skin pigmentation or nasal cavity blockage. Notably, both nasal cavities were identical in size, and there was no family history of such disorders. Importantly, the patient had not undergone therapy for the mass previous to this appointment and reported no associated comorbidities.

**Figure 1:**
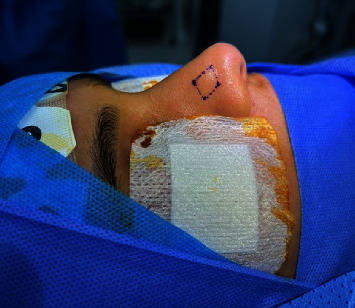
Nasal examination revealed external right nasal swelling at right ala extended from right lower lateral cartilage up to right upper lateral cartilage.

A general physical checkup revealed no abnormalities. The neurological examinations were within normal. A nasal examination revealed an external enlargement on the right nasal ala, which extended from the right lower lateral cartilage to the right upper lateral cartilage (Figure 1). The tumor was spherical, cystic, movable, and nontender and showed no redness or infection. Lymph node examination was unremarkable.

**Figure 2:**
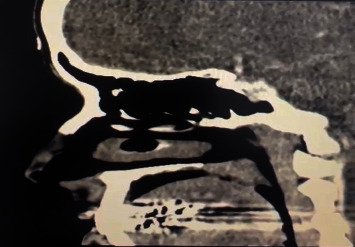
Computed tomography demonstrating isolated poorly defined soft tissue swelling within the right-sided lateral nasal wall anteriorly. There was a mild heterogeneous contrast enhancement but no clear expansion to surrounding structures.

**Figure 3:**
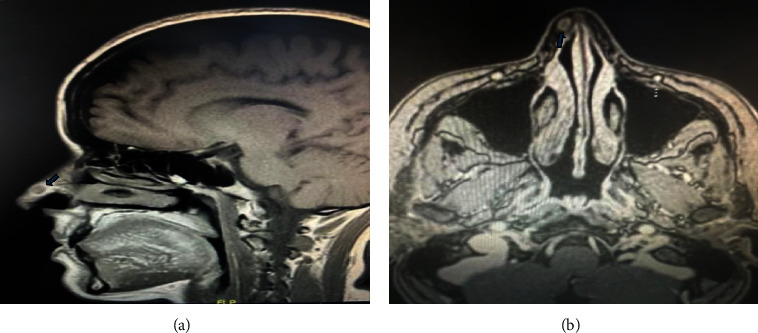
Sagittal (a) and axial (b) magnetic resonance imaging confirmed the presence of an oval lesion (arrows) measuring about 0.5 x 0.7 cm with isointense signals in all pulse sequences, with homogenous postcontrast enhancement, clear surrounding fat planes with no invasion to nasal septum or other adjacent structures.

A contrast-enhanced computed tomography (CT) was done, demonstrating isolated poorly defined soft tissue swelling within the right-sided lateral nasal wall anteriorly. There was a mild heterogeneous contrast enhancement but no clear expansion to surrounding structures (Figure 2). Further assessment with magnetic resonance imaging (MRI) confirmed the presence of an oval lesion approximately 0.5 x 0.7 cm in size, with isointense signals across all pulse sequences and homogenous postcontrast enhancement. The tumor was surrounded by distinct fat planes, with no invasion of the nasal septum or any nearby structures (Figure 3). The MRI also revealed a modest increase in the size, signal intensity, and enhancement patterns when compared to a previous MRI performed in 2018, providing a baseline for assessing the growth rate. The surgical option, including potential risks and advantages, was reviewed with the patient, who later agreed to the operation.

## 3. Operative Procedure

**Figure 4:**
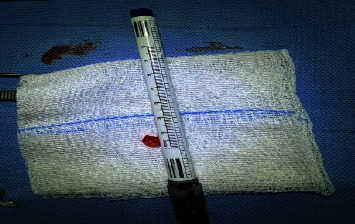
The mass was successively dissected from the surrounding structures and excised completely.

The patient underwent open rhinoplasty with excision of the right nasal mass under general anesthesia. A midcolumellar incision at the waist of the nasal *columella* was made, followed by bilateral marginal incisions. A sub-SMAS dissection technique was utilized, and the nasal skin flap was extended laterally to achieve adequate exposure of the mass. Operative findings were consistent with the radiological impressions. The mass was meticulously dissected from the surrounding structures and completely excised (Figure 4). The excised tissue was sent for histopathological examination. Closure was achieved using monocryl 5−0 sutures for the transcolumellar and marginal incisions, respectively, preserving all major supports of the nasal tip. Postoperative management included nasal taping and splint application. The patient's recovery was uneventful, with satisfactory aesthetic and functional outcomes, leading to her discharge with appropriate home medications.

**Figure 5:**
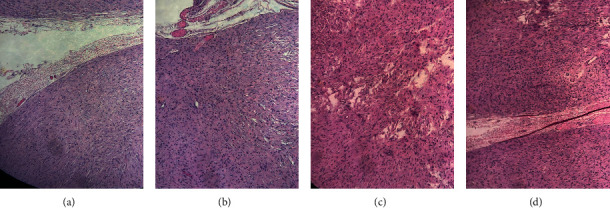
Histopathology of the specimen revealed (a) a well-circumscribed spindle cell neoplasm, showing vague storiform pattern with collagenized stroma. (b) Cells have indistinct cytoplasm and wavy nuclei. (c, d) Mild pleomorphism with no increase in mitosis or necrosis.

Postoperative histopathological analysis of the excised specimen was suggestive of a peripheral nerve sheath tumor (PNST) (Figure 5).

## 4. Follow-up

The patient was followed up regularly in our outpatient clinic for eight months postsurgery. During each visit, we performed two-point discrimination testing around the mass, she was evaluated for any signs of recurrence or complications, and none were observed. The patient reported significant improvement in her quality of life and satisfaction with the cosmetic results of the surgery.

## 5. Discussion

Peripheral nerve sheath tumors (PNSTs), which develop from Schwann cells in association with peripheral nerve axons and dendritic cells, encompass both benign and malignant forms. Benign PNSTs, including schwannomas and neurofibromas, represent 10–12% of benign soft tissue neoplasms [9, 10], whereas malignant PNSTs are the fifth most common type of malignant soft tissue neoplasms [9, 11, 12]. Malignant forms such as neurofibrosarcoma and malignant schwannoma may arise sporadically or from malignant transformation of neurofibromatosis type 1, occasionally following radiotherapy [2, 6, 13]. Notably, 25%–45% of extracranial benign PNSTs occur in the head and neck region; however, their presence in the nasal dorsum and tip is exceedingly rare [8].

Symptoms of nasal PNSTs vary depending on their location within or on the nose. Although often asymptomatic [15–17], these tumors can cause nasal obstruction, bleeding, rhinorrhea, ptosis, and facial swelling [18, 19]. Neurological symptoms might manifest when the mass compresses or involves the nerve of origin; for example, facial nerve involvement can lead to facial palsy, while trigeminal nerve involvement typically remains asymptomatic [12, 13, 20].

Differential diagnosis for nasal masses is broad, ranging from pleomorphic adenoma to angiofibroma [21]. Given the nonspecific clinical presentation of PNSTs, imaging techniques such as CT scans and MRI are critical to assess the size and extent of the mass and to help exclude conditions like encephalocele or intranasal glioma [3, 8, 18, 22]. These imaging modalities also aid in distinguishing between benign and malignant masses based on characteristics such as growth rate and bone interaction [13, 23].

Histological examination remains essential for confirming the diagnosis [18]. For benign PNSTs, surgical excision is the preferred treatment, balancing both cosmetic and oncological outcomes [4, 8, 18]. In this regard, an open rhinoplasty approach is particularly advantageous for tumors located on the nasal dorsum or tip, as evidenced by multiple case studies [24–26]. Prognostically, malignant PNST outcomes hinge on factors such as early detection, tumor size, and the completeness of excision [6, 27].

## 6. Conclusion

Our study is limited by its single-case format, which may not provide a comprehensive view of the variability in clinical presentations and outcomes of PNSTs. Future research should focus on larger cohort studies to better understand the biological behavior of PNSTs and refine diagnostic and therapeutic approaches. Additionally, exploring the molecular mechanisms of malignant transformation in neurofibromatosis-associated PNSTs could offer insights into targeted therapies and preventative strategies.

## Data Availability

The data used to support the findings of this study are available upon request.
